# Contraception for midlife women: lack of information? Lack of interest? Lack of investment?

**DOI:** 10.1186/s40695-017-0025-7

**Published:** 2017-09-13

**Authors:** Nancy Fugate Woods, Gloria Bachmann

**Affiliations:** 1 Biobehavioral Nursing and Health Informatics, University of Washington, Seattle, WA USA; 2 Obstetrics, Gynecology and Reproductive Sciences, Robert Wood Johnson Medical School, Piscataway Township, NJ USA

## Abstract

Despite the growing population of midlife women in the USA and in other economically-advantaged countries, there is scant research focusing on this population, especially regarding contraceptive choices in those at risk for unwanted or unplanned pregnancy. As women approach menopause, the increasing irregularity of their menstrual cycles and changing endocrine patterns present challenges for preventing unintended pregnancy, or, on the flip side, planning for a desired pregnancy.

In this thematic series, we have brought together researchers and clinicians who address many of these tough clinical scenarios and offer suggestions not only for clinical management of optimal contraceptive choices for midlife women, but also suggestions for future research in this area.

Despite the growing population of midlife women in the USA and in other economically-advantaged countries, there is scant research focusing on this population, especially regarding contraceptive choices in those at risk for unwanted or unplanned pregnancy. As women approach menopause, the increasing irregularity of their menstrual cycles and changing endocrine patterns present challenges for preventing unintended pregnancy, or, on the flip side, planning for a desired pregnancy.

One wonders why there is a dearth of interest in studying the contraceptive challenges that both women and their health care providers face when deciding on the best options for the midlife period in the lifespan (variously defined as 35, 40 or 45 to 65 years of age, or women born between 1952 and 1982). There are some midlife women who are not sexually active, sexually active only with female partners, or not interested in contraception, while others believe they are already infertile or do not contemplate the risk of an unintended pregnancy. In this thematic series, we have brought together researchers and clinicians who address many of these tough clinical scenarios and offer suggestions not only for clinical management of optimal contraceptive choices for midlife women, but also suggestions for future research in this area.

Those women who do wish to prevent unintended pregnancy confront many limitations. Among these limitations are the lack of information about midlife women’s preferences for different types of contraceptives. For example, would long-acting reversible contraceptives (LARC) be optimal? Do midlife women prefer injectables or intrauterine systems? Do midlife women still desire to experience menses or prefer to avoid menstruating? Do midlife women rely on sterilization of their sexual partners or do they opt for tubal ligation after their last pregnancy? Do health care providers assume that midlife women need hormone replacement instead of contraception? Do some contraceptives, like the progestin-containing IUD, actually assist women who are experiencing extremely distressing vasomotor symptoms [1] in commencing estrogen therapy without the need for an added systemic progestin?

One could ask if the lack of interest in national contraceptive policy for midlife women is due to the low fertility of this age group of women, despite its high risk of maternal morbidity, mortality, and high unintended pregnancy rates. Or is this gap just a function of a lack of interest by investigators and health policy scholars in the health of midlife women?

What is known about different subsets of women? We are not all the same! Some midlife women desire pregnancy. What is their preferred choice of contraception to protect their future fertility? What is the optimal choice for them? The subset of midlife women who no longer desire pregnancy have contraceptive options, but how do they make a decision about the best option for them and when is it appropriate for them to consider a permanent method? For women in heterosexual relationships, what counseling should they receive regarding vasectomy for their male partner? And what are the health care costs associated with each choice?

Information is particularly scant for midlife transgender men—who have maintained their uterus, fallopian tubes and ovaries—and who are in a sexual relationship with male partner. Are providers assisting them with contraceptive options that do not bring about unwanted feminizing effects, but still offer adequate contraceptive protection?

Midlife is a period during which women need to consider health risks related to use of various types of contraception. During midlife, women begin to be at increased risk for chronic diseases, such as hypertension and diabetes. What data exist to guide their choices? As we will see in the articles in this issue, many questions that women, health care providers, and researchers raise are answered without validation by robust empirical data.

Contemporary midlife women have been socialized about sex, contraception and family planning and the option of pregnancy termination differently from those who are a decade or two older or younger. Baby Boomers, some of whom are among the older portion of midlife women, were among the first to receive some (albeit not comprehensive) sex education in the US school system. They may have been among the first cohorts of women to have access to oral contraceptives, as well. In addition, they have had access to legal abortion since the 1973 Roe v Wade decision by the US Supreme Court. The oldest of this group experienced their reproductive years in the midst of the early days of the feminist movement, during which women were socialized to exercise choices with which they felt comfortable and were not restricted to options imposed upon them or strongly suggested to them by significant others or family members. Most were exposed to the changing roles and opportunities for women in society at large, and thus made decisions about when and if to become pregnant. They also experienced motherhood by choice, either through pregnancy or adoption.

At the same time, however, midlife women were not necessarily exposed to better contraceptive education. In fact, interest in research about midlife was limited until the early 1990s when investigations for studies such as the Study of Women and Health Across the Nation (SWAN), the Penn Ovarian Aging Study, and the Seattle Midlife Women’s Health Study were launched in an effort to generate better understanding of the hormonal changes, symptoms, and health of midlife women in the context of their contemporary lives.

Despite these research endeavors, the incongruity of thinking about contraception and reproductive aging minimized the focus of these studies on contraception and unintended pregnancy, especially for the population of women during the later years of their natural reproductive life cycle.

In all countries — regardless of income status — national, international and donor policies related to contraception and family planning services have important implications for the health of midlife women. Research to advance understanding of optimal approaches to family planning that includes a full range of clinical services for women is particularly critical at this life-stage, as is the inclusion of midlife women in eligibility criteria for receipt of contraceptive and family planning programming, and careful assessment of costs and other barriers that may prevent midlife women from accessing to services.

Today, professional health care practices for midlife women, at their best, emphasize the most effective methods to prevent pregnancy. Clinicians place less emphasis on selecting a contraceptive method in the context of chronic illness, or on consideration of women’s personal fertility plans. These remain topics that have not been universally integrated into contraceptive counseling. This thematic series of *Women’s Midlife Health* provides a strong voice and a strong commencement to addressing this important topic of contraception in midlife women.
